# Validation of a microwave radar system for the monitoring of locomotor activity in mice

**DOI:** 10.1186/1740-3391-4-7

**Published:** 2006-05-04

**Authors:** Vittorio Pasquali, Eugenio Scannapieco, Paolo Renzi

**Affiliations:** 1 Dipartimento di Psicologia, Sezione di Neuroscienze, Università di Roma "La Sapienza", Via dei Sardi 70, 00185 Roma, Italy

## Abstract

**Background:**

The general or spontaneous motor activity of animals is a useful parameter in chronobiology. Modified motion detectors can be used to monitor locomotor activity rhythms. We modified a commercial microwave-based detection device and validated the device by recording circadian and ultradian rhythms.

**Methods:**

Movements were detected by microwave radar based on the Doppler effect. The equipment was designed to detect and record simultaneously 12 animals in separate cages. Radars were positioned at the bottom of aluminium bulkheads. Animal cages were positioned above the bulkheads. The radars were connected to a computer through a digital I/O board.

**Results:**

The apparatus was evaluated by several tests. The first test showed the ability of the apparatus to detect the exact frequency of the standard moving object. The second test demonstrated the stability over time of the sensitivity of the radars. The third was performed by simultaneous observations of video-recording of a mouse and radar signals. We found that the radars are particularly sensitive to activities that involve a displacement of the whole body, as compared to movement of only a part of the body. In the fourth test, we recorded the locomotor activity of Balb/c mice. The results were in agreement with published studies.

**Conclusion:**

Radar detectors can provide automatic monitoring of an animal's locomotor activity in its home cage without perturbing the pattern of its normal behaviour or initiating the spurt of exploration occasioned by transfer to a novel environment. Recording inside breeding cages enables long-term studies with uninterrupted monitoring. The use of electromagnetic waves allows contactless detection and freedom from interference of external stimuli.

## Background

The general or spontaneous motor activity of animals is a useful parameter in chronobiology. Since this type of research generally requires a large amount of data from several weeks of monitoring, the use of automatic systems is necessary.

Various types of automatic systems to measure the locomotor activity of rodents can be found in the literature: the most common ones are activity wheels [1], capacity condensers [2], Doppler effect systems [3], stabilimeters [4], ultrasound recorders [5], touchplate recorders [6,7], infrared recorders [8], video-tracking systems [9] and telemetry systems [10]. Critical evaluation of the monitoring systems shows that they must fulfil the following criteria: 1) the behaviour to be recorded must be clearly defined; 2) the animal's activity must not be affected by the structure of the monitoring apparatus; 3) the sensitivity of the apparatus must be uniform in space; 4) the recording technique must not be intrusive; 5) the monitoring must be continuous and automatic; 6) the output must be non-stop and easy to analyse, preferably with a computer; 7) the apparatus must have a simple calibration method so that its sensitivity is replicable and stable over time; and 8) the apparatus must be validated by comparison of its output with the same activity recorded in another way, preferably by manual recording of the observations.

Radar-based monitoring systems have proved effective in the study of behaviour, both in very small animals like insects [11] and in small mammals [12]. Radar systems have various advantages (for details, see [11]), especially the possibility to monitor the animal in its breeding cage, which is very important in pharmacological studies or in research on stress factors.

The aim of the present study was to validate an apparatus for the monitoring and recording of locomotor activity in mice. The apparatus is based on an electronic recording system designed and tested by our research group [13] but subsequently subjected to a new series of more rigorous tests. The apparatus, named VIVARD-12, permits the monitoring of general motor activity of 12 mice housed individually in standard breeding cages.

## Methods

### Electronic system for the recording of locomotor activity

The locomotor activity of the animal is recorded automatically by means of microwave radar based on the Doppler effect. Microwave radar systems operate at the frequency of 9.9 GHz (Mw-12, Lince Italia Srl), with a wavelength of around 3 cm. The sensitivity is normally controlled by a trimmer with a narrow regulation range (22 kohm). We replaced the component with a 100 kohm trimmer to obtain a finer regulation scale and better control of the circuit's sensitivity. The high-frequency electromagnetic emissions produced by the radar device have a power of around 10 mW·cm^-2^, which does not interfere with the animal's behaviour. The radar devices were connected to a computer via a digital I/O card (PIO-12, Keithley Instruments). The incoming signals were also diverted to an LED that signalled the recording of movement with an impulse of +5 VDC. A simple program, written in C language (Micaloni, Renzi, Pasquali), continually read the channels of the I/O card. All the parameters – sampling frequency of 10-2,000 msec, collected interval of the given datum (rate at which the accumulated counts are saved to disk, in seconds or minutes), length of the experiment (minutes or days) – are easily modifiable via the program. The number of radar devices supported by the computer is strictly dependent on the number of channels of the I/O card. The following controls were carried out: a) interference between adjacent radars, b) setting of the sensitivity (76 kohm – with this value the radar responded only to movement of the whole body and not to any of its parts alone), c) measurement of the same number of movements, d) temporal stability of the settings, and e) lack of signal emissions in the absence of movement. All the radar devices were set up with the aid of a mechanical object with standardized movement.

### Structure of the apparatus

**Figure 1:**
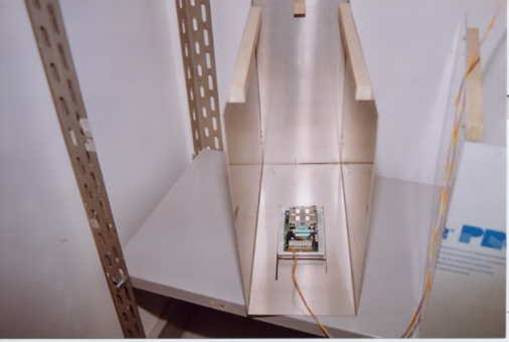
Positioning of the radar device at the bottom of the aluminium frame.

**Figure 2:**
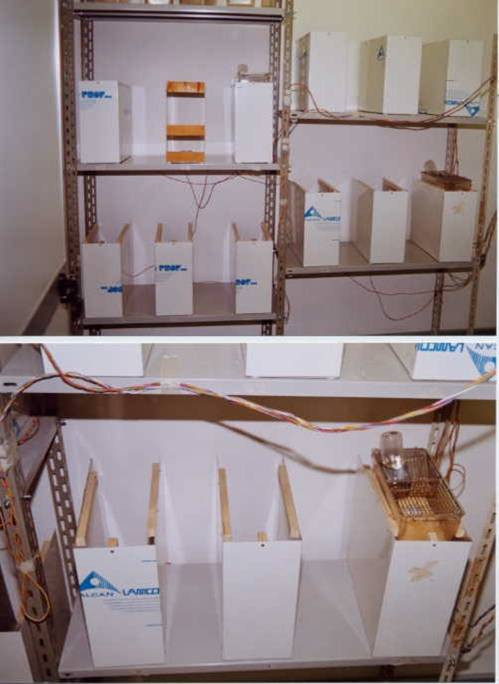
The complete apparatus.

The apparatus was designed for the simultaneous monitoring and recording of 12 individually housed animals. Each radar device was positioned at the bottom of an aluminium structure (17 × 36 × 40 cm) that supported the animal's cage, screened the radar device from possible interference by nearby radars, and assured perfect alignment of the cage with respect to the coverage area of the radar (Figure 1). The alignment was determined by several pieces of wood attached to the aluminium structure. The aluminium structures were positioned on plain metal shelves to further isolate the radar devices situated at different levels (Figure 2). The radar devices were connected to the computer's data acquisition card by a multipolar electric cable (single wire, 1 mm diameter) that was intertwined to improve the shielding against electromagnetic fields.

The apparatus was evaluated by several tests using both mechanical objects with standardized movement and laboratory animals.

### Test 1

The aim of the first test was to verify the ability of the apparatus and the subsequent computer analyses to record the exact frequency of movement of an object with standardized movement. For this purpose, we used the second hand of a clock whose frequency was 1 movement per minute.

## Materials

**Figure 3:**
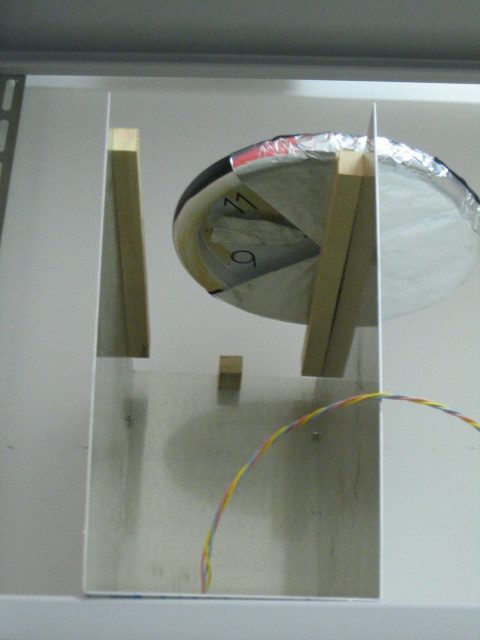
Position of the clock and acquisition window.

A Wellgain wall clock with second hand was positioned on top of the aluminium structure, where the animal's cage was usually lodged. A radar reflector consisting of a piece of aluminium (3 × 6 cm) was attached to the second hand. An aluminium protection with a window corresponding to 1/4 of a full rotation was fixed in front of the area of rotation of the second hand (Figure 3). The window was positioned exactly on the perpendicular of the radar's recording cone; thus, the second hand was visible (i.e., in movement) for only 15 seconds each minute. In this way, we obtained an object with a frequency of movement of once per minute.

### Procedure

Twelve 24-hour recordings were performed, i.e. one for each radar device. The parameters of the software were: sampling frequency = 500 msec, collected interval = 3 seconds, and length of the experiment = 1440 minutes (1 day). For each time series, the data were accumulated in 30-sec bins. Fourier analysis was then applied to determine the rhythmicity in the recording.

## Results and discussion

**Figure 4:**
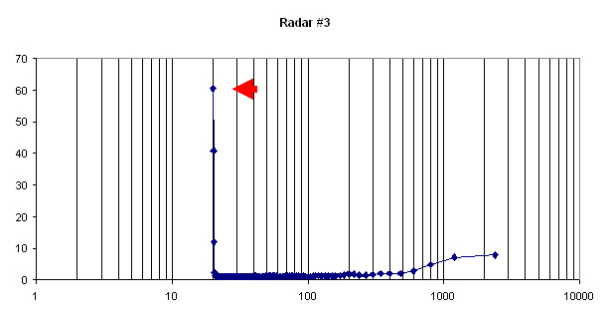
**Spectral analysis of data from Test 1**. The peak at 20 bins corresponds to 60 seconds (1 bin = 3 sec). Power values on the y-axis; x-axis is periods (in seconds) in logarithmic scale.

All the spectra showed a peak corresponding to a frequency of 1 movement per minute (Figure 4). The power of the rhythm was often different and other rhythmicities could be observed in the spectra. This could have been due to the recording system, i.e. a loss of stability of the measurements of the recording devices. However, it was probably caused by the gear mechanism of the clock: not being very precise, it may have had different frictions and inaccuracies during the rotations. Therefore, we carried out a second test to evaluate the temporal stability of the sensitivity of the radar devices.

### Test 2

A fundamental characteristic of a monitoring system is stability in time, i.e. the measurements must remain constant. The setting of the apparatus must not change with time or with use. Therefore, we designed a test to evaluate this condition and to re-check the spurious rhythmicities observed in test 1.

## Materials

**Figure 5:**
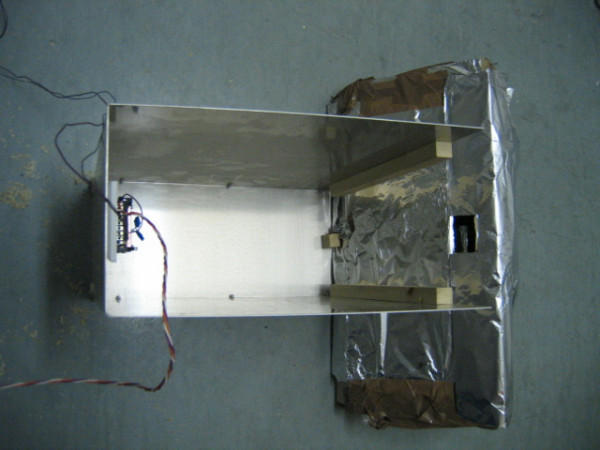
Screening of the metronome and position of the apparatus and acquisition window.

For the second test, we positioned a Wittner metronome on top of the apparatus. A radar reflector consisting of a piece of aluminium (3 × 2.5 cm) was glued to the apex of the pendulum. The minimum oscillation of the metronome was 1 oscillation per second, i.e. 60 oscillations per minute. The entire metronome was placed in a cardboard container, completely closed except for a square hole (3.5 × 3.5 cm) (Figure 5). The hole faced the radar, and the periodic passage of the piece of aluminium was visible through the hole.

### Procedure

We performed 90-minute recordings for 6 of the 12 radar devices. The software parameters were: sampling frequency = 500 msec, collected interval = 30 seconds, and length of the experiment = 90 minutes. To evaluate the constancy in time of the recordings of each device, we considered the recordings as consisting of three 30-minute parts. For the analysis of variance of the data, we considered the three parts of the recording as the three experimental conditions and the 30-second recordings as the single cases.

## Results and discussion

There were no significant differences in the number of movements counted in any of the cases. This indicates that the sensitivity of each radar device was uniform throughout the 90-minute period (Table 1).

**Table 1:** Means of the three parts of the recording interval and statistical results.

**RADAR 2**		**RADAR 5**	
	
17.02	F (2,177) = 0.27, p = 0.7671	12.80	F (2,177) = 0.95, p = 0.3878
16.85		13.27	
16.83		12.78	
**RADAR 12**		**RADAR 9**	
	
18.53	F (2,177) = 0.44, p = 0.6416	16.27	F (2,177) = 0.83, p = 0.4382
18.93		16.13	
18.78		15.80	
**RADAR 10**		**RADAR 11**	
	
15.07	F (2,177) = 2.5, p = 0.0851	15.22	F (2,177) = 0.83, p = 0.4359
15.18		15.55	
14.67		15.48	

### Test 3

After the tests using objects with standardized movement, we performed behavioural tests with animals. The purpose was to determine what types of movements the radar effectively detects and to evaluate the sensitivity of the radar to the different movement classes.

## Materials

Two male mice belonging to the C57BL/6 strain (Charles Rivers Laboratory, Calco, Como, Italy) were housed individually in 369 × 156 × 132 (h) mm Plexiglas cages, with a light:dark (L:D) 12:12 photoperiod, a constant temperature of 21°C, and water and food ad libitum.

**Figure 6:**
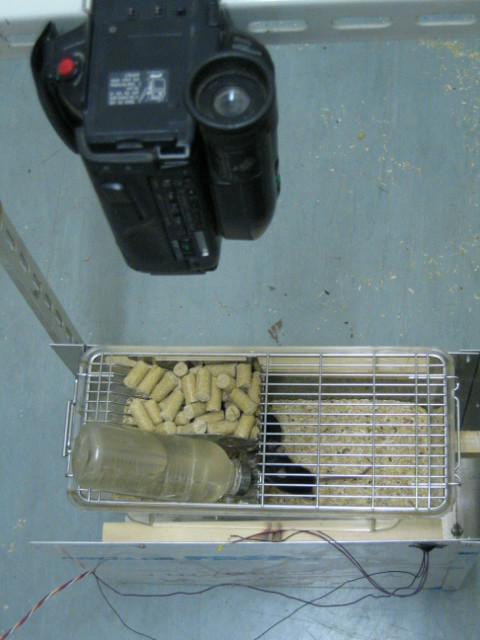
Setup for video-recording of the animal.

Each animal was video-recorded with a Sony Handycam videocamera situated 30 cm above the cage (Figure 6). An LED was connected to the radar and placed in the field of the videocamera but outside the visual field of the mouse. The LED lit up when the radar recorded movement.

### Procedures

Each animal was video-recorded for 8 hours. From the video playback, we analysed the mouse's activity for 1 minute of each 10-minute period for a total of 48 minutes. The following behavioural categories were established, and we determined if the radar recorded them when the mouse performed them:

• locomotion (walking, running, jumping);

• climbing (hanging or climbing on the bars of the cage, with two or four paws);

• digging (the sawdust is moved forward or backward with the head or the front limbs);

• drinking/eating/biting the cage (the animal stands upright and licks the bottle, chews the food, bites the bars);

• grooming (rubbing, cleaning, licking the face, fur, ears, tail, genitals);

• rising on two legs/lowering onto all four legs;

• turning (rotating the anterior part of the body while remaining on both hind limbs);

• broad head movements;

• stretching;

• scratching the fur with the front paws.

The recordings were examined independently by two observers.

## Results and discussion

Observation of the animals' activities and the simultaneous lighting of the LED showed that the radar devices are very sensitive to movements involving a shift of the whole body (Table 2). The other behavioural categories were recorded in a lower percentage of cases.

**Table 2:** Agreement between the behavioural categories recorded by the human observers and those recorded by the radar device.

	Observer 1	Observer 2	Observer 1	**Mean**
	radar7	radar7	radar6	
	
LOCOMOTION	100%	99%	99%	**99.3 %**
CLIMBING	100%	93%		**96.5 %**
STRETCHING	100%	100%	100%	**100.0 %**
DIGGING	100%	85%	59%	**81.3 %**
RISING/LOWERING	100%	91%	86%	**92.3 %**
TURNING	98%	78%	87%	**87.7 %**
BITING	0%	0%		**0 %**
DRINKING/EATING	0%	0%	0%	**0 %**
GROOMING	13%	46%	7%	**22.0 %**
SCRATCHING	37%	50%	25%	**37.3 %**
HEAD MOVEMENTS	54%	63%	44%	**53.7 %**

### Test 4

In the fourth test, we recorded and analysed the locomotor activity of mice whose locomotor parameters have been well described, i.e. amount of activity, length of the circadian period, and strength of circadian rhythmicity (as indicated by the spectral power of the circadian period).

## Materials

We used 10 8-week-old male mice of the Balb/c strain (Charles Rivers Laboratory; Calco, Como, Italy). The mice were housed individually with food and water ad libitum, L:D 12:12 (lights on 8–20), temperature of 21 ± 1°C and humidity of 55 ± 5 %.

### Procedure

The mice were housed individually in 369 × 156 × 132 (h) mm Plexiglas cages. After three days, we began the 28-day period of video-recording: the first week in LD 12:12 and the next three weeks in DD. For the behavioural analyses, we only considered the 7 days in LD 12:12 and the last 7 days in DD. The recordings were carried out in a sound-proof, air-conditioned room.

### Data analysis

All the time series were detrended and treated with a three-point moving mean procedure. The treated series were then analysed with discrete Fourier transform [14] to obtain information in the domain of frequencies. The output of the Fourier analysis was initially analysed with the Kolmogorov-Smirnov test for comparison with a random distribution of the peaks. For series significantly different from a random distribution (all of them), only the peaks with power greater than 2.88 standard deviations from the mean were subsequently considered significant (p < 0.001). To estimate the circadian period, we analysed the data with the periodogram of Sokolove and Bushnell [15], as implemented by Refinetti [16], testing the periods between 20 and 26 hours. The data for the number of movements and the length and spectral power of the circadian period were tested by ANOVA.

## Results and discussion

**Figure 7:**
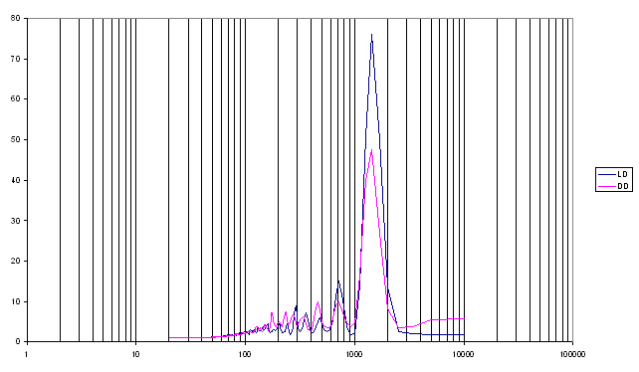
**Spectral analysis of data from Test 4**. Power values on the y-axis; x-axis is periods (in minutes) in logarithmic scale.

We determined the level of activity of each animal in terms of number of signals counted by the software. The mice showed a significant difference in the length of the circadian period in LD and DD (23.98 vs. 23.04 hours) [t(18) = 17.33, p < 0.001]. However, neither the spectral power of the circadian period (76.0 vs. 47.4) nor the amount of activity (139175 vs. 116815) differed significantly between the two conditions, even though they decreased in DD. Finally, spectral analysis showed the presence of ultradian rhythms with several significant peaks in the range 1–8 hours and a main peak at 12 hours (Figure 7).

These results agree with literature reports that the Balb/c strain has an endogenous, genetically determined circadian period that is shorter than 24.0 hours [17-19]. The other two parameters were also comparable to those reported in the literature, particularly the reduced spectral power of the circadian peak in DD [19-21]. Therefore, the monitoring system can reliably record the various parameters of locomotor activity.

## Conclusion

The aim of this study was to develop an apparatus consisting of a battery of radar sensors to allow the investigation of mouse activity rhythms. In addition, we wanted to re-validate the locomotor monitoring system that our research group designed and validated several years ago. The new apparatus allows easier recording of animals by means of a battery of radar devices housed in specific elements and arranged in a smaller space with respect to the old system.

Unlike the first validation study [13], the present tests were not based on comparison with another apparatus but on the ability of the monitoring system to identify the frequencies and rhythms of motion of objects with standardized movement. Moreover, we carried out tests with mice belonging to inbred strains whose behavioural parameters are genetically determined and well known, particularly the length of the endogenous circadian period.

In general, our system was able to record the exact rhythmicity of the moving object in test 1 and to perform constant counts in time. Moreover, the results for the recorded behavioural categories agree with previous reports of "general locomotor activity" and of the activity parameters of the Balb/c strain; in particular, the circadian period is consistent with the results of many studies in the literature. The recorded ultradian periodicities are also consistent with the few data present in the literature [22-29]. In fact, the study of ultradian rhythms appears to be particularly difficult, since it is necessary to identify short rhythms that have wide variability by means of mathematical algorithms, and the monitoring system must not create masking effects or influence the normal behaviour of the animal. For example, a commonly used instrument, the running wheel, tends to affect the activity patterns of rodents and must be considered an active recording system that masks the endogenous structure of the rhythms expressed by the animal, especially ultradian rhythms [30].

We believe that our monitoring system is particularly suitable for the study of activity rhythms in mice. The use of electromagnetic waves does not interfere with the animal's behaviour, and the animals can be left in their breeding cages, thus avoiding a change of environment and the resulting changes in exploratory activity [31]. The computerized recording system also permits very long monitoring of the animals, creating continuous time series. The data files are automatically saved to the hard disk, allowing immediate analyses of the data.

## Competing interests

The author(s) declare that they have no competing interest.

## Authors' contributions

VP and ES carried out the experiments and prepared the initial draft of the manuscript. PR supervised the experiments and produced the final version of the manuscript. The study was conceived and planned by VP. VP and ES contributed equally to the work. All authors approved the final version of the manuscript.
